# A new statistical approach to training load and injury risk: separating the acute from the chronic load

**DOI:** 10.5114/biolsport.2024.127388

**Published:** 2023-07-19

**Authors:** Lena Kristin Bache-Mathiesen, Thor Einar Andersen, Torstein Dalen-Lorentsen, Montassar Tabben, Karim Chamari, Benjamin Clarsen, Morten Wang Fagerland

**Affiliations:** 1 Oslo Sports Trauma Research Centre, Department of Sports Medicine, Norwegian School of Sport Sciences, Oslo, Norway; 2 Department of Smart Sensors and Microsystems, SINTEF Digital, Oslo, Norway; 3 Aspetar, Orthopaedic and Sports Medicine Hospital, FIFA Medical Centre of Excellence, Doha, Qatar; 4 Centre for Disease Burden, Norwegian Institute of Public Health, Bergen, Norway; 5 Department of Health and Functioning, Faculty of Health and Social Sciences, Western Norway University of Applied Sciences, Bergen, Norway; 6 Oslo Centre for Biostatistics and Epidemiology, Research Support Services, Oslo University Hospital, Oslo, Norway

**Keywords:** Training monitoring, Load monitoring, Soccer, ACWR, acute:chronic

## Abstract

The relationship between recent (acute) training load relative to long-term (chronic) training load may be associated with sports injury risk. We explored the potential for modelling acute and chronic loads separately to address current statistical methodology limitations. We also determined whether there was any evidence of an interaction in the association between acute and chronic training loads and injury risk in football. A men’s Qatar Stars League football cohort (1 465 players, 1 977 injuries), where training load was defined as the number of minutes of activity, and a Norwegian elite U-19 football cohort (81 players, 60 injuries), where training load was defined as the session rating of perceived exertion (sRPE). Mixed logistic regression was run with training load on the current day (acute load) and cumulative past training load estimated by distributed lag non-linear models (chronic load) as independent variables. Injury was the outcome. An interaction between acute and chronic training load was modelled. In both football populations, we observed that the risk of injury on the current day for different values of acute training load was highest for players with low chronic load, followed by high and then medium chronic load. The slopes varied substantially between different levels of chronic training load, indicating an interaction. Modelling acute and chronic loads separately in regression models is a suitable statistical approach for analysing the association between relative training load and injury risk in injury prevention research. Sports scientists should also consider the potential for interactions between acute and chronic load.

## INTRODUCTION

Researchers attempt to identify risk factors for sports injuries to protect the athletes’ health and improve sport performance [1]. One potential, modifiable risk factor is training load. Training load is the mechanical, physiological and psychological load resultant of multiple episodes of physical activity performed by an athlete [2]. Hypotheses suggest that not only high or low training load levels may affect injury risk, but also rapid increases in recent training load relative to training load incurred in the past [3]; i.e. a peak in the relative training load [3].

Hulin, Gabbett [4] introduced the Acute:Chronic Workload Ratio (ACWR) to estimate the effect of relative training load on the risk of sports injury [3, 5]. In their model, the most recent training load, the acute load, is divided by the past, or chronic load. In theory, the higher the ratio – the higher the acute load relative to the chronic – the higher the risk of injury [3]. After ACWR became popular, concerns were raised on its theoretical and methodological foundations [6]. Among others: the number of subjective choices involved increased risk of spurious findings due to multiplicity issues [7], the time lengths for the acute and chronic periods were arbitrary [8], and it could not handle an acute or chronic load of 0 [6].

A core principle in the theory underlying the ACWR is that the effect of the acute load depends on the amount of chronic load. If acute load is high, it may not necessarily increase injury risk if the chronic load is also high. The aim of the ACWR was therefore to adjust the acute load to the chronic load, estimating the effect of acute load properly. This adjustment is not always successful when calculating a ratio [6, 9]. Instead, Wang, Vargas [10] suggested modelling the acute load and the chronic load separately. This eliminates the risk that acute load will not be properly adjusted to the chronic load. At the time of Wang et al.’s proposal, several other challenges remained unsolved, including how to estimate the cumulative effect of past training load, the chronic load. Recent research suggests this may be solved by applying the distributed lag non-linear model [11].

The theory that the effect of acute load depends on the level of chronic load suggests an interaction between acute and chronic loads. Previous descriptive research has studied the association of ACWR with injury for different chronic loads [12, 13], but none have so far modelled an interaction between acute and chronic loads outside of the ACWR framework. Whether an interaction can be assessed while chronic load is modelled by distributed lag non-linear model is also unknown. Distributed lag non-linear models can explore time-lagged effects, but it cannot determine what time period is considered “recent” and “past” in the context of relative training load [11].

We hypothesized that exposure to training affects injury risk on the current day, but the training *stimuli* on the current day does not contribute to injury risk on that day. In contrast, the accumulated stimuli (fitness) built on past training days *does* contribute to injury risk on the current day. In addition, if the athlete does not participate in training on the current day, the athlete is obviously not at risk on that day [14]. We argue that the current day of training is therefore markedly different from past training days, and it may thus be possible to consider the current day only as the acute load, and all past observations as chronic load.

Investigating whether there is evidence of an interaction between acute and chronic loads association with injury risk may elucidate whether such interactions are worth considering in future research, and whether they are possible to model using distributed lag non-linear models.

The primary aim of this statistical methodology study was to demonstrate how modelling acute and chronic training loads separately can be used to describe an association between relative training load and injury risk – meant for use in training load research. A secondary aim was to find out whether acute and chronic loads interact in their association with injury risk in football.

## MATERIALS AND METHODS

### Participants

We analysed eight competitive seasons (2015–2022) from the men’s Qatar Stars League injury surveillance registry in football (1 465 players, 1 977 injuries, Supplemental Table S1), and one season from a Norwegian elite U-19 football cohort (81 players [45% female], 81 injuries) described in Dalen-Lorentsen, Andersen [7].

### Ethics

The Anti-Doping Lab Qatar Institutional Review Board approved the Qatar Stars League study (E2017000252). The Aspire Zone Foundation Institutional Review Board approved a data sharing agreement between Aspetar Orthopaedic and Sports Medicine Hospital and Oslo Sports Trauma Research Centre. The Norwegian Center for Research Data (5487), and the South-Eastern Norway Regional Committee for Medical and Health Research Ethics (2017/1015), approved the Norwegian elite U-19 study. Ethical principles were followed in accordance with the Declaration of Helsinki, and informed consent was obtained from all participants.

### Training load definition

In the Qatar Stars League data (1 136 223 observations, 12% missing data), training load was defined as the daily number of minutes in activity (football training, other training, and/or match-play).

In the Norwegian elite U-19 data (8 494 observations, 24% missing data), training load was defined as: the daily number of minutes of activity (football training, other training, and/or match-play), multiplied by the player’s rating of perceived exertion on a scale from 0 to 10, deriving the session Rating of Perceived Exertion (sRPE) [15].

Missing data were imputed using multiple imputation (Supplemental Figure S1–S2) [16, 17].

### Injury definition

Injuries in Qatar Stars League players were recorded prospectively using the Sport Medicine Diagnostic Coding System classification [18, 19]. We recorded all injuries that reduced training or match play participation (time-loss injuries). The player was considered injured until the team medical staff allowed full training and match participation. We did not record injuries that occurred outside football activities. Quality control was performed to ensure injury validity (Supplementary). Injuries were classified as either sudden or gradual onset.

The Norwegian elite U-19 players reported daily whether they had experienced a new health problem, with Briteback AB online survey platform, Norrköping, Sweden. If they had, a clinician conducted a structured interview and classified the health problem as being an injury or an illness according to the Union of European Football Associations guidelines [20]. Only injuries were analysed in this study. Injury definitions in both populations followed the 2006 consensus statement on epidemiological studies in football [21].

### Statistical analysis Simple model example

To demonstrate how acute and chronic loads can be modelled separately to study the relationship between relative training load and injury risk, we performed a simple statistical analysis on the Qatar Stars League data that mimicked traditional methodological choices in the training load and injury risk field.

We ran a logistic regression with injury yes/no as the outcome. The acute load and chronic loads were two independent variables in the model. Acute load was the sum of the current week of training (minutes in activity). Chronic load was the average daily minutes in activity in the concurrent 3 weeks before the acute load week, calculated with the exponentially weighted moving average [22]. The analysis thus represents the so-called uncoupled 1:3 ACWR, which has been recommended over the coupled ACWR [23]. However, instead of calculating a ratio, the acute and chronic loads were modelled as separate independent variables.

We caution that the assumptions of this simple analysis, such as linearity, are unlikely to be met [24, 25].

### Advanced statistical approach

The main analysis of this study was an advanced statistical model run to meet two aims: (i) To demonstrate how to model acute and chronic loads separately in an advanced statistical framework, (ii) To test whether there is an interaction between acute and chronic loads’ association with injury risk in football.

To estimate the association of relative training load with the risk of injury, a logistic mixed model was run, with injury yes/no as the outcome. A random intercept per player accounted for the possibility that some players are inherently more likely to suffer injuries than others [26]. We denote the model run on the Qatar Stars League data the Qatari model, and the model run on the Norwegian elite U-19 data the Norwegian model.

The independent variables in the model were the acute and the chronic loads. Choice of acute and chronic time windows should be based on hypothesis/rationale or prior evidence [3, 27]. Given our rationale in the introduction and elaborated upon in the discussion, we considered the acute load to be the current day of training (Day 0).

The relationship between the acute load and injury risk might be non-linear [28], and therefore we applied restricted cubic splines with 3 knots [25]. The knot locations were based on the range of the training load observations in the Qatar Stars League data (Qatari model) and the Norwegian elite U-19 data (Norwegian model), respectively; subjectively placed knots have shown improved performance over data-driven placement on skewed training load distributions [25].

Chronic load was the training performed during the previous 27 days, excluding day 0. Day -1 is the day before the current day (yesterday), Day -2 two days before the current day, and so on up to Day -27, which is 27 days before the current day (four weeks ago). We assumed that training load values closer to the current day contribute more to injury risk than those distant in time [22]. We also assumed that the association between training load and injury may be different depending on the time since the activity [3]. For example, if hypothetically, 60 minutes of activity three weeks ago decreases risk of injury, while 60 minutes of activity performed yesterday increases risk of injury, we aimed to be able to detect that difference. Therefore, the cumulative effect of chronic load was modelled with a distributed lag non-linear model [11]. This approach estimates the association between training load and the risk of injury, and simultaneously estimates how the association with training load changes depending on the time since the activity. We chose restricted cubic splines to model the association with training load (3 knots), and also restricted cubic splines to model the association with number of days since the activity was performed (4 knots).

An interaction term was added between the acute load (Day 0) and the chronic load (Day -1 to day -27). The main result was a visualization of the predicted probabilities of injury for acute load given different levels of chronic training load. Reference levels of chronic load was chosen by finding examples of zero, low, medium and high chronic load in the original data (Supplemental Table S2).

Since players are only at risk of injury if they participate in an activity, days in which they did not participate in any training or match were removed from the analysis. These observations were still included in the estimation of chronic load.

To see if a simpler approach than distributed lag non-linear model can be suitable, the analyses were repeated using the exponentially weighted moving average on chronic load [22].

Additional analyses were performed on the Qatar Stars League data. First, the Qatari model was performed on sudden – and gradual-onset injuries, separately [18]. Second, we explored the risk of injury for various levels of minutes in activity sustained in the past, using the distributed lag non-linear model.

Statistical analyses were performed in R (4.2.1) with DLNM [29], mice, lme4, and slider [30]; code available online [31].

## RESULTS

### Simple model example

In the logistic regression, odds ratios (OR) were estimated for the acute load (0.995) and chronic load (1.016) separately (Table 1).

**TABLE 1:** The risk of injury in Qatar Stars League football players estimated by a logistic regression model.

Parameter^1^	OR	SE	95% CI	p
Intercept	0.005	0.0004	0.004–0.006	< 0.001
Acute load	0.995	0.0002	0.994–0.995	< 0.001
Chronic load	1.016	0.0012	1.014–1.019	< 0.001

Abbreviations: CI = Confidence Interval; OR = Odds Ratio; SE = Standard Error

1 Acute load was defined as the current week of training (sum of minutes in activity), while chronic load was defined as the 3 weeks of training prior to the acute week (exponentially weighted moving average [EWMA] of daily minutes in activity)

**FIG. 1:**
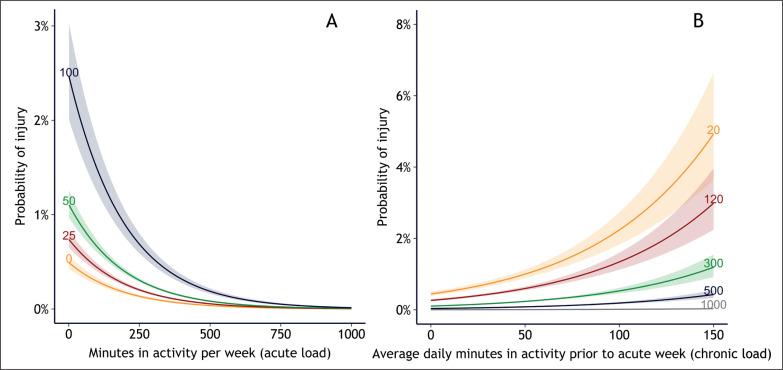
The probability of injury for each level of (A) acute load and (B) chronic load in Qatar Stars League players (564 206 exposure values, 1 006 injury cases). In (A), the risk is shown for different levels of chronic load: 0, 25, 50, and 100 average minutes in activity the previous 3 weeks. In (B), the risk is shown for different levels of acute load: 20, 120, 300, 500, and 1000 minutes in activity in the current week.

This allowed further investigation into the risk of injury for each level of acute load, given the level of chronic load (Figure 1A), and vice versa (Figure 1B). The results showed an increased risk for each decrease in acute load, and increased risk for each increase in chronic load. For example: A player with 20 minutes of training in the current week, who had 150 minutes daily training the previous performed (the W-function). Since F was modelled with 3 knots, and W with 4, the result is a 3*4 permutation of intervals 3 weeks, had 5% increased injury probability, while a player who trained 300 minutes on the current week had 1% increased injury probability, despite having the same amount of chronic load (Figure 1B). This is a common pattern when injured players reduce loads the remaining week, thus have lower loads than uninjured players [32]. Since the ORs were of similar size, this indicates that the acute load (which stretches over just one week) may be more important than the chronic load (which stretches over three weeks).

### Association between training load and injury risk

**FIG. 2:**
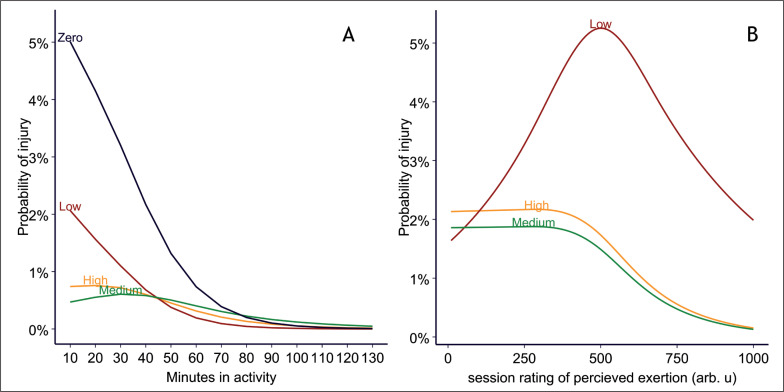
Estimated probability of injury for each level of acute load (the current day) for (A) Qatari model (420 329 exposure values, 1 977 injuries) and (B) Norwegian model (4 719 exposure values, 60 injuries). The probability is shown for zero, low, medium and high chronic load levels; these are defined in Supplemental Table S1. Due to multicollinearity in the data, confidence intervals could not be estimated. Arb. u = arbitrary units.

In the main analysis, the acute load was defined as the load on the current day (day 0), and chronic load was defined as the load during the past 27 days (day -1 to day -27). The Qatari model showed decreased probability of injury for each minute in activity on the current day (acute load) with statistical significance (p < 0.001, Figure 2, Table 2). This is a typical pattern when players end activity early due to injury. Players who had not participated in an activity in the last 27 days were at highest risk of injury, followed by those who spent a low number of minutes in activity (Figure 2A). Players who spent a high number of minutes in activity were at higher risk than those with medium (Figure 2A). Some relationship slopes were steep, other slopes were gradual, and this variation suggests an interaction between number of minutes in activity on the current day and the minutes in activity the previous 27 days (Figure 2A). All of the 12 interaction terms had narrow confidence intervals (Table 2). This interaction was also present in both sudden onset and gradual onset injuries (Figure S5).

**TABLE 2:** QSL model cofficients for a logistic regression with injury as the outcome and minutes in activity on the current day (acute), and past minutes in activity (chronic) as independent variables.

Term^123^	OR	SE	Lower CI	Upper CI	P
Intercept	0.067	0.329	0.034	0.131	< 0.001
Acute minutes in activity 1	0.950	0.007	0.936	0.964	< 0.001
Acute minutes in activity 2	1.144	0.017	1.105	1.185	< 0.001
Chronic minutes in activity W1 F1	2.166	0.252	1.282	3.659	0.006
Chronic minutes in activity W1 F2	0.455	0.129	0.348	0.595	< 0.001
Chronic minutes in activity W1 F3	1.285	0.110	1.030	1.602	0.027
Chronic minutes in activity W2 F1	0.156	0.374	0.075	0.324	< 0.001
Chronic minutes in activity W2 F2	6.112	0.191	4.207	8.881	< 0.001
Chronic minutes in activity W2 F3	0.841	0.181	0.590	1.200	0.340
Chronic minutes in activity W3 F1	3.252	0.623	0.952	11.109	0.060
Chronic minutes in activity W3 F2	0.578	0.363	0.279	1.198	0.137
Chronic minutes in activity W3 F3	0.673	0.281	0.388	1.168	0.159
Chronic minutes in activity W4 F1	6.432	1.228	0.578	71.55	0.130
Chronic minutes in activity W4 F2	0.319	0.642	0.090	1.126	0.076
Chronic minutes in activity W4 F3	0.404	0.573	0.130	1.256	0.116
Interaction (Acute*Chronic minutes W1 F1)	0.998	0.003	0.991	1.006	0.642
Interaction (Acute*Chronic minutes W1 F2)	1.002	0.002	0.998	1.006	0.429
Interaction (Acute*Chronic minutes W1 F3)	1.000	0.001	0.998	1.003	0.844
Interaction (Acute*Chronic minutes W2 F1)	1.020	0.004	1.012	1.028	< 0.001
Interaction (Acute*Chronic minutes W2 F2)	0.978	0.002	0.974	0.982	< 0.001
Interaction (Acute*Chronic minutes W2 F3)	1.004	0.002	1.001	1.008	0.020
Interaction (Acute*Chronic minutes W3 F1)	0.993	0.006	0.982	1.005	0.243
Interaction (Acute*Chronic minutes W3 F2)	1.010	0.003	1.003	1.017	0.009
Interaction (Acute*Chronic minutes W3 F3)	1.003	0.003	0.997	1.009	0.340
Interaction (Acute*Chronic minutes W4 F1)	0.996	0.009	0.978	1.015	0.678
Interaction (Acute*Chronic minutes W4 F2)	1.005	0.005	0.995	1.015	0.311
Interaction (Acute*Chronic minutes W4 F3)	1.007	0.005	0.997	1.016	0.154

Abbreviations: CI = 95% Confidence Interval, OR = Odds Ratio, QSL = Qatar Stars League, SE = Standard Error

^1^All variables were modelled with splines (420 329 exposure values, 1 977 injuries), and terms represent one of multiple intervals demarcated by knots

^2^The DLNM models a cross-product of the number of minutes in activity (the F-function) and the lag time in which the activity was performed (the W-function). Since F was modelled with 3 knots, and W with 4, the result is a 3*4 permutation of intervals

A similar pattern was displayed in the Norwegian model: low chronic sRPE increased risk of injury, followed by high, with the lowest risk at medium levels of chronic sRPE (Figure 2B). Also, like the Qatari model, the Norwegian model exhibited major changes in the slopes between the different levels of chronic sRPE, indicating an interaction (Figure 2B). However, the model failed to estimate coefficients for certain spline intervals on the chronic load (Table S3).

The relationship shape between the training load variables did not change by including random effects (Figure S3), and some of the coefficients were inestimable in the mixed model. Therefore, random effects were not included in the final models.

The additional models, where chronic load was calculated with the exponentially weighted moving average, failed to discover an association between chronic training load and injury risk (Figure S4).

**FIG. 3:**
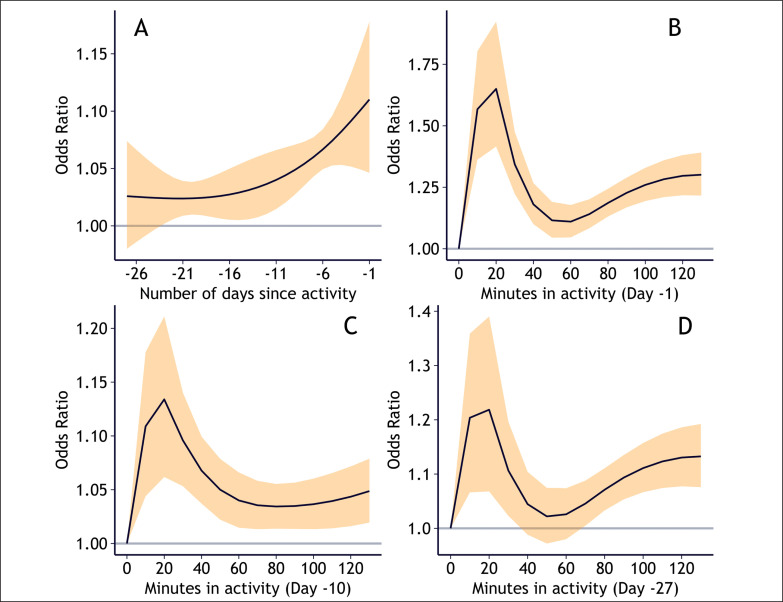
Injury risk profiles of chronic load in Qatar Stars League players (1 136 223 exposure values, 1 977 injuries). Panel A shows the risk of 60 minutes of activity for each day in the past: -1 is the risk of injury if the activity occurred the day prior to the current day, and -27 is the risk if the activity occurred 27 days before the current day. Panels B, C, and D shows how the risk of injury changes for each level of minutes in activity if the activity occurred (B) 1 day prior to the current day, (C) 10 days prior to the current day, (D) 27 days prior to the current day. Y-axes for B–D are not on the same scale, to better show the relationship shape. Yellow bands represent 95% confidence intervals.

In the additional model exploring how the relationship between training load and injury risk changes with time on the Qatar Stars League data, activities performed on the day before the current day (day -1) contributed most to the risk of injury on the current day (OR = 1.1 for 60 minutes of activity, 95% confidence interval (CI) = 1.05–1.18, Figure 3). The risk declined exponentially the more distant in time the activity was performed, ending at approximately OR = 1.02 (CI = 1.01–1.04) for 60 minutes of activity performed 19 to 22 days prior to the current day. A low number of minutes in activity (10–40 minutes) on a day in the past substantially increased risk of injury for the current day, a high number (90–120 minutes) moderately increased risk, and a medium number (40–80 minutes) slightly increased risk, regardless of whether the activity was performed 1 day prior to the current day, 10 days prior, or 27 days prior (Figure 3B–D).

## DISCUSSION

This is the first study to explore the potential of modelling acute and chronic training loads separately to estimate the association between relative training load and injury risk in sport. In a simple model example, where traditional definitions of acute and chronic load was used, the new statistical approach provided separate effect estimates for the acute, current week of training (OR = 0.995, 95% CI = 0.994–0.995) and the chronic, previous three weeks of training (OR = 1.016, 95% CI = 1.014–1.019).

In our main analysis, where acute load was defined as the current day and chronic load as the previous 27 days (4 weeks), acute load (minutes in activity) was associated with the probability of injury in Qatar Stars League football (Qatari model). This was properly adjusted for the cumulative association with chronic load. Signs of an association between acute load (sRPE) and injury risk could also be gleaned in a Norwegian elite U-19 football population (Norwegian model), although with high uncertainty due to a small sample size.

We also investigated whether there was an interaction between acute and chronic training loads. Evidence of an interaction was found, as in both the Qatari and the Norwegian models, the relationship slopes for acute training load varied considerably for different levels of chronic training load.

### Modelling acute and chronic loads separately

Modelling the acute and chronic load separately successfully estimated the association of acute load adjusted for the levels of chronic load, in both our simple model example and the more advanced statistical model (main analysis). This investigation did not require calculating a ratio, a method that has been severely critiqued [6, 33]. One advantage of modelling acute and chronic separately over the ACWR is that analysts can determine which time period, acute or chronic, is more important concerning injury risk. In addition, while using the ACWR would require choosing among multiple ways of calculation [7], the current approach required few such choices, and reduced the risk of multiple testing issues.

In the main analysis, low chronic training load displayed highest risk, followed by high chronic load, then medium chronic load with the lowest risk, in both the Qatari and Norwegian model. In addition, having zero chronic load the last four weeks (a month without football) showed the highest risk of injury in the Qatari model. Importantly, this could not have been discovered if we had used any form of ratio, as the denominator would be 0 [9].

### Association and interaction between acute and chronic loads in football

The Qatari model indicated decreased injury risk for each minute spent in activity on the current day (p < 0.001). The Norwegian model displayed a similar trend, although non-significant (p > 0.05), and injury risk increased if chronic load (cumulative past sRPE) was low. We suspect that players who ended activity due to an injury skewed the models toward decreased risk with increased exposure to training load. This effect was amplified in the Qatari model, which only included time-loss injuries and time in exposure – no measure of the training intensity. This is a general and – yet – unsolved challenge for studies that aim to estimate the association between training load and injury risk.

Interestingly, the slopes of the association between chronic load and injury risk varied considerably in the Qatari model: High and medium chronic load slowly declined in risk for each level of acute load, while low chronic load declined rapidly (Figure 2A). This interaction was also present when stratified on sudden onset and gradual onset injuries. In the Norwegian model, low chronic load both increased and decreased risk at different levels of acute load (Figure 2B). Therefore, to improve injury prevention research, we would recommend future training load and injury risk studies consider and explicitly model these interactions.

The model that estimated chronic load with the exponentially weighted moving average failed to discover an association between chronic load and injury risk. Given the large sample size of the Qatar Stars League data, we speculate whether this approach could estimate the effects at all, even in a larger study.

The distributed lag non-linear model allowed exploration of time-lagged effects between chronic load and injury risk [11]. In the Qatar Stars League population, the risk of injury declined exponentially for each day further back in time the activity was performed. Furthermore, a low number (10–40) or a high number (80–120) of minutes in activity on a day in the past both increased risk of injury on the current day, while a medium number (40–80 minutes) decreased risk in comparison. This fits the hypotheses that both too much and too little training may increase risk of injury [34].

### Background for considering acute load as the current day of training

A consistent challenge with traditional methods of estimating relative training load’s effect on injury risk is choosing the time periods for acute and chronic load [8, 35]. Subjectively deducing the cut-off may be arbitrary [35], cut-offs based on previous research may not be sport-specific [6], and data-driven approaches risk multiple testing issues and reduced comparability [32].

We hypothesized that the current day (Day 0) has special properties compared to past days of training load exposure, which allows it to be modelled separately.

On the current day, injury risk increases with sheer exposure to the physical activity itself. Players cannot sustain an injury if they do not participate in an activity [3]. On the other hand, if players did not participate in an activity on certain days in the past, those days would still contribute to the cumulative effect of past training load. Thus, the effect of a training load value of 0 changes drastically if it is on the current day versus past training load days.

Hypotheses suggest that both high and low levels of training load may increase injury risk [34]. Too little training will not build enough fitness for the tissue to tolerate upcoming training load levels. Too much training may potentially damage the tissue, and the tissue may not regenerate in time for the next training or match-play exposure. These hypotheses pertain mostly to past training load. On the current day, the player enters with fitness and fatigue resultant of the past. The adaptations built during the current day of training will not likely come into play until later (that day or during the successive days). The fatigue, will, however affect the current training and day. Hence, the shape of the relationship between training load and injury risk (linear, or various non-linear), may depend on whether the event was in the past, or on the current day.

In a real-time setting, the current and future days of training or match-play load are the most modifiable. One cannot change training load that happened in the past. Team sports coaches develop training schedules for a year, for a month, but most importantly for a week ahead, often according to the match schedule, in so-called training micro-cycles [36]. Weekly risk estimates cannot inform how training load should be distributed on each day within a week or micro-cycle [24], but daily risk estimates can. Studies interested in causal inference and developing load management programs should take this into consideration when choosing time periods for acute and chronic loads.

### Future perspectives

In this paper, we have showed the potential of modelling acute and chronic training loads separately. While this study focused on football, we believe the proposed method can handle sport-specific circumstances, such as tapering, and can be considered for both individual and team sports. In addition, although this study only assessed associations, this statistical approach can be used in studies of causal inference or prediction, given that methodological considerations for each of the respective study aims are taken into account [37]. Finally, our simple model example shows that an advanced approach is not needed to model acute and chronic loads separately. It can be used with any choice of time periods for acute and chronic loads, which is particularly relevant for studies that only have access to data at a weekly level.

Distributed lag non-linear modelling is a flexible approach to handling the complexity of chronic load. The R-package was, however, developed in epidemiology, and not yet adapted to interactions. Future research is needed in implementation of distributed lag non-linear models for the context of training load.

### Limitations

Limitations of this study were: (i) Due to multicollinearity in our data, confidence intervals around predictions in Figure 2 could not be estimated, (ii) the Qatar Stars League data only had minutes of activity, and no other training load variables or variable describing the intensity of the activity; (iii) the Norwegian elite U-19 data had only sRPE – the player’s perception of the training exertion and the duration of the activity. Different groups of players can perceive the same physiological stimuli differently [38]; the Norwegian elite U-19 sRPE responses were above other football populations [39, 40]. In this regard, training load is a multidimensional construct, and ideally, both internal and external training loads should be used [2].

## CONCLUSIONS

To assess the association between recent (acute) training load relative to past long-term (chronic) training load on injury risk, a ratio has traditionally been calculated. Ratios have several challenges and cannot handle chronic loads of 0. Modelling the acute and the chronic load separately is intuitive and potentially a simple solution to this problem. When using this statistical approach, the acute load adjusts for the level of chronic load without calculating a ratio. Furthermore, signs of an interaction between acute and chronic training load were present in both football populations studied. Researchers in the field of training load and injury risk should consider interactions in their respective sport to improve injury prevention research.

## Supplementary Material

A new statistical approach to training load and injury risk: separating the acute from the chronic load
