# Supplementary material for: A new statistical approach to training load and injury risk: separating the acute from the chronic load
Source: Biol Sport. 2023 Jul 19;41(1):119–34. doi: 10.5114/biolsport.2024.127388 (PMC10765439; doi:10.5114/biolsport.2024.127388)
Supplement: A new statistical approach to training load and injury risk: separating the acute from the chronic load [file JBS-41-50672-s1.pdf]

English Premier League Soccer Players. International Journal of Sports Physiology and Performance. 2015; 10(4):489–97. doi: 10.1123/ijspp.2014-0352.
37. Shmueli G. To explain or to predict? Statistical science. 2010; 25(3):289–310. doi: 10.1214/10-STS330.
38. Impellizzeri FM, Rampinini E, Coutts AJ, Sassi A, Marcora SM. Use of RPE-based training load in soccer. Medicine & Science in Sports & Exercise. 2004; 36(6):1042–7. doi: 10.1249/01.MSS.0000128199.23901.2F.
39. Rabbani A, Clemente FM, Kargarfard M, Chamari K. Match Fatigue Time-Course Assessment Over Four Days: Usefulness of the Hooper Index and Heart Rate Variability in Professional Soccer Players. Frontiers in Physiology. 2019; 10(109). doi: 10.3389/fphys.2019.00109.
40. Chamari K, Haddad M, Wong DP, Dellal A, Chaouachi A. Injury rates in professional soccer players during Ramadan. Journal of Sports Sciences. 2012; 30(sup1):S93–S102. doi: 10.1080/02640414.2012.696674.

SUPPLEMENTARY

Injury validation in Qatar Stars League registry

The team physician in each club was in charge of collecting the data, using standardized tools. We distributed a study manual outlining the details of data collection to the contact person before the team's enrollment into the study. We also organized demonstration sessions every time a new team physician joined the program. We recorded data using a custom-made Microsoft Office Excel® file (Microsoft Corporation, Readmon, WA, USA) for quick data entry, using pull-down menus to classify each injury based on the Sport Medicine Diagnostic Coding System. Injury cards were also provided in Microsoft Office Word® (Microsoft Corporation, Readmon, WA, USA) to assist clinicians in taking notes during daily clinical activity, prior to entry into the master data file. We asked the clubs to submit their data every month by email. Data quality control was done on a monthly basis to validate the data.

TABLE S1. Characteristics of 1 465 Qatar Stars League players for the 3 365 studied player' seasons.

**Characteristic**[1]	**Mean (SD)**
Age (n = 564)	25 (5)
Height (n = 535)	174 (21)
Weight (n = 548)	71 (16)
Player position (n = 725)[2]	**N (%)**
Defenders	231 (32%)
Goal Keepers	81 (11%)
Midfielders	316 (44%)
Strikers	97 (13%)

[1]Variables had missing data, and descriptives are calculated on observed values (n).
[2]One player could change positions across multiple seasons, and therefore be included multiple times in the calculation.

Articles published in the Biology of Sport are licensed under an open access Creative Commons CC BY 4.0 license.

TABLE S2. Chronic load profiles used as reference values in Figure 1 (main article), Figure S3 and Figure S5, from the day before the current day (-1) to 27 days prior to the current day (-27).

	Qatar Stars League[1]				Norwegian elite U-19[2]		
**Day**	**Zero**	**Low**	**Medium[3]**	**High[4]**	**Low**	**Medium[3]**	**High[4]**
-1	0	60	90	45	80	480	720
-2	0	60	27	45	0	0	630
-3	0	60	79	90	0	720	540
-4	0	0	60	80	0	588	1260
-5	0	0	30	80	0	120	0
-6	0	0	63	80	0	0	560
-7	0	0	30	90	0	450	0
-8	0	0	63	140	0	30	0
-9	0	0	60	105	0	0	1230
-10	0	0	11	70	0	540	0
-11	0	0	78	40	0	900	810
-12	0	0	15	15	0	390	0
-13	0	0	77	45	0	90	0
-14	0	0	13	45	0	240	0
-15	0	0	78	90	0	370	320
-16	0	0	75	90	0	30	0
-17	0	0	0	90	0	360	0
-18	0	0	0	90	0	60	0
-19	0	0	70	90	0	0	0
-20	0	0	70	90	0	540	0
-21	0	0	70	45	0	55	630
-22	0	0	26	90	0	0	360
-23	0	0	70	30	0	0	0
-24	0	0	70	45	0	0	960
-25	0	0	70	45	0	30	360
-26	0	0	70	90	0	540	0
-27	0	0	70	45	0	630	420
Total	0	180	1435	1900	80	7163	8800

[1] Measured in minutes in activity
[2] Measured in session Rating of Perceived Exertion (sRPE) in arbitrary units
[3] The total sum was the median in the corresponding dataset
[4] The total sum was the 75% quantile in the corresponding dataset

TABLE S3. Model coefficients for a logistic regression with injury as the outcome and sRPE on the current day (acute), and past sRPE (chronic) as independent variables in the Norwegian elite U-19 data.

Term[123]	OR	SE	Lower CI	Upper CI	p
Intercept	0.035	1.110	0.004	0.308	0.003
Acute sRPE	1.001	0.003	0.996	1.006	0.656
Acute sRPE	0.997	0.002	0.992	1.001	0.177
Chronic sRPE W1 F1	0.111	1.055	0.014	0.883	0.038
Chronic sRPE W1 F2	0.972	0.660	0.266	3.544	0.965
Chronic sRPE W1 F3	2.661	0.638	0.758	9.343	0.126
Chronic sRPE W2 F1	369558.600	4.787	30.843	4.43E+09	0.007
Chronic sRPE W2 F2	0.122	2.538	0.001	17.66	0.407
Chronic sRPE W2 F3	0.230	2.724	0.001	48.581	0.589
Chronic sRPE W3 F1	0.000	15.939	0.000	390.613	0.108
Chronic sRPE W3 F2	13.162	6.383	0.000	3647113	0.686
Chronic sRPE W3 F3	4.529	6.798	0.000	2924533	0.824
Chronic sRPE W4 F1	0.000	33.76	0.000	0.324	0.046
Chronic sRPE W4 F2	22218.120	13.56	0.000	8.02E+15	0.461
Chronic sRPE W4 F3	92.306	15.116	0.000	8.11E+14	0.765
Interaction (Acute*Chronic sRPE W1 F1)	1.005	0.002	1.001	1.009	0.016
Interaction (Acute*Chronic sRPE W1 F2)	1.000	0.001	0.997	1.002	0.866
Interaction (Acute*Chronic sRPE W1 F3)	0.999	0.001	0.996	1.001	0.259
Interaction (Acute*Chronic sRPE W2 F1)	0.971	0.009	0.954	0.988	0.001
Interaction (Acute*Chronic sRPE W2 F2)	1.005	0.005	0.996	1.014	0.310
Interaction (Acute*Chronic sRPE W2 F3)	0.999	0.005	0.990	1.009	0.900
Interaction (Acute*Chronic sRPE W3 F1)	1.056	0.026	1.003	1.111	0.038
Interaction (Acute*Chronic sRPE W3 F2)	0.989	0.014	0.962	1.016	0.418
Interaction (Acute*Chronic sRPE W3 F3)	1.008	0.012	0.984	1.033	0.500
Interaction (Acute*Chronic sRPE W4 F1)	1.161	0.057	1.039	1.298	0.009
Interaction (Acute*Chronic sRPE W4 F2)	0.967	0.030	0.912	1.025	0.262
Interaction (Acute*Chronic sRPE W4 F3)	1.017	0.027	0.964	1.074	0.535

Abbreviations: CI = 95% Confidence Interval, OR = Odds Ratio, SE = Standard Error, sRPE = session Rating of Perceived Exertion in arbitrary units

[1] All variables were modelled with splines, and terms represent one of multiple intervals demarcated by knots

[2] The DLNM models a crossproduct of the number of minutes in activity (the F-function) and the lag time in which the activity was performed (the W-function). Since F was modelled with 3 knots, and W with 4, the result is a 3*4 permutation of intervals

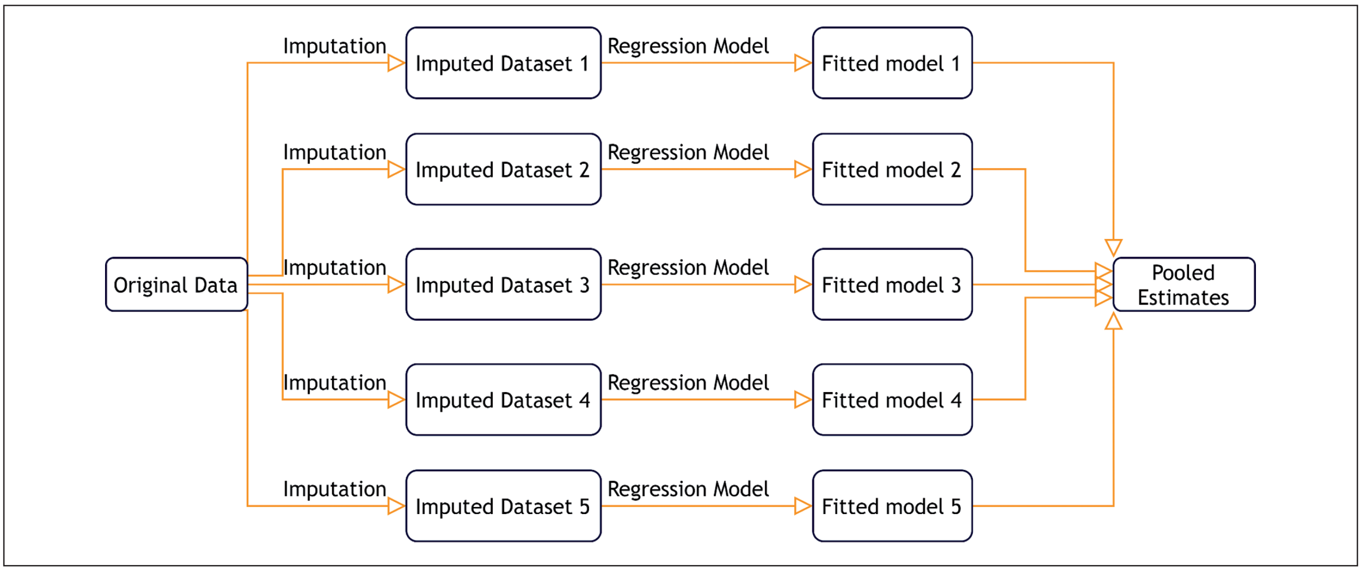

FIG. S1. Illustration of the modelling process in the framework of multiple imputation. The imputation was performed in accordance with recommendations in "Flexible Imputation of Missing Data, Second Edition" by Stef van Buuren (Van Buuren, 2018a), also available online (Van Buuren, 2018b). Missing time in activity in minutes, and sRPE values, were predicted and imputed using predictive mean matching (Barzi & Woodward, 2004), which has previously been shown to be a valid approach for count data (Van Buuren, 2018a). For the minutes in activity, a poisson regression imputation was compared with the PMM with validation plots, before choosing PMM. All non-derived variables were used to predict imputed values, including age, sex, player position, type of training activity, among others. The response variable, injury, was also used to predict imputed values (Moons et al., 2006), but was not itself imputed before analysis (Peters et al., 2012). The number of imputed datasets was five, which is recommended in most cases (Van Buuren section 2.8). The imputation was validated by comparing the distribution of the imputed versus the original data (see Figure S2). Five models were fitted and pooled using Ruben's Rules for the final models (results in Table 1 and Table 2, main article).

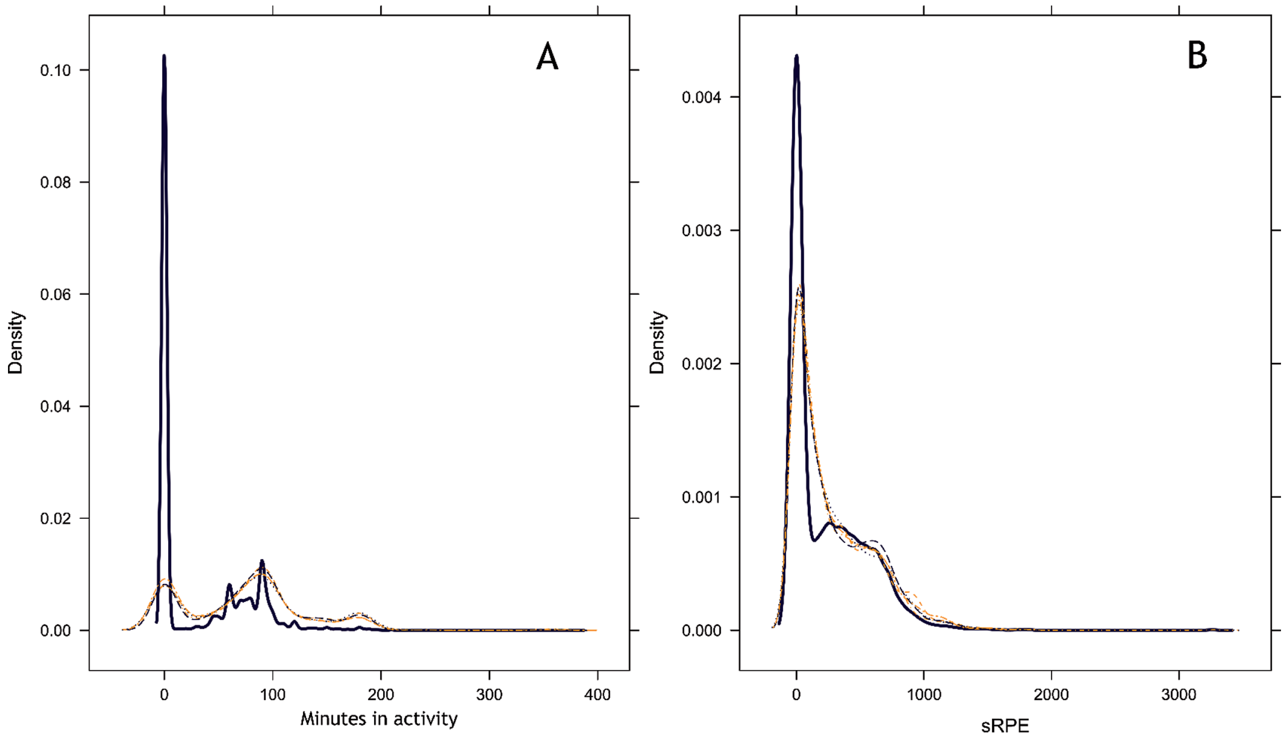

FIG. S2. Distribution of original data values (blue) compared to imputed values from five imputed datasets (yellow) for (A) daily minutes in activity in a Qatar Stars League football population, and (B) daily session Rating of Perceived Exertion (sRPE) measured in arbitrary units in a Norwegian elite U-19 football cohort. The mismatch between the distribution of imputed data and original data in (A) is expected. Although 12% of the Qatar Stars League exposure observations were missing, on days that players suffered an injury, the missing rate was 36%. The missing mechanism was therefore missing at random, and missing probability increased if injury = yes. Since players are unlikely to be injured on days with no activity (exposure = 0), one would expect the imputed distribution to skew less towards 0 than the original data.

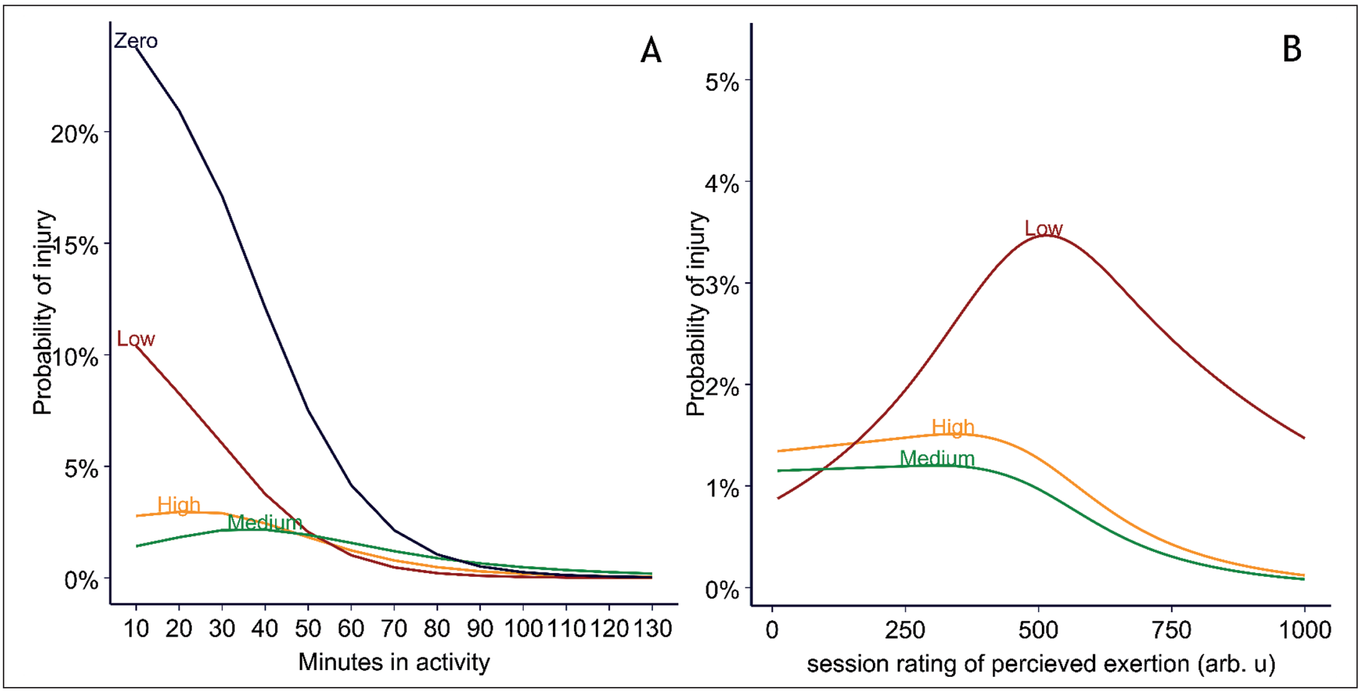

FIG. S3. Probability of injury on the current day (Day 0) predicted by logistic regression models with random effects. Shown for each level of training load variables used in (A) Qatar Stars League model (420 329 exposure values, 1 977 injuries) and (B) Norwegian elite U-19 model (4 719 exposure values, 60 injuries). The probability is shown for zero, low, medium and high cumulative chronic training load levels, as defined in Table S2. Arb. u = arbitrary units.

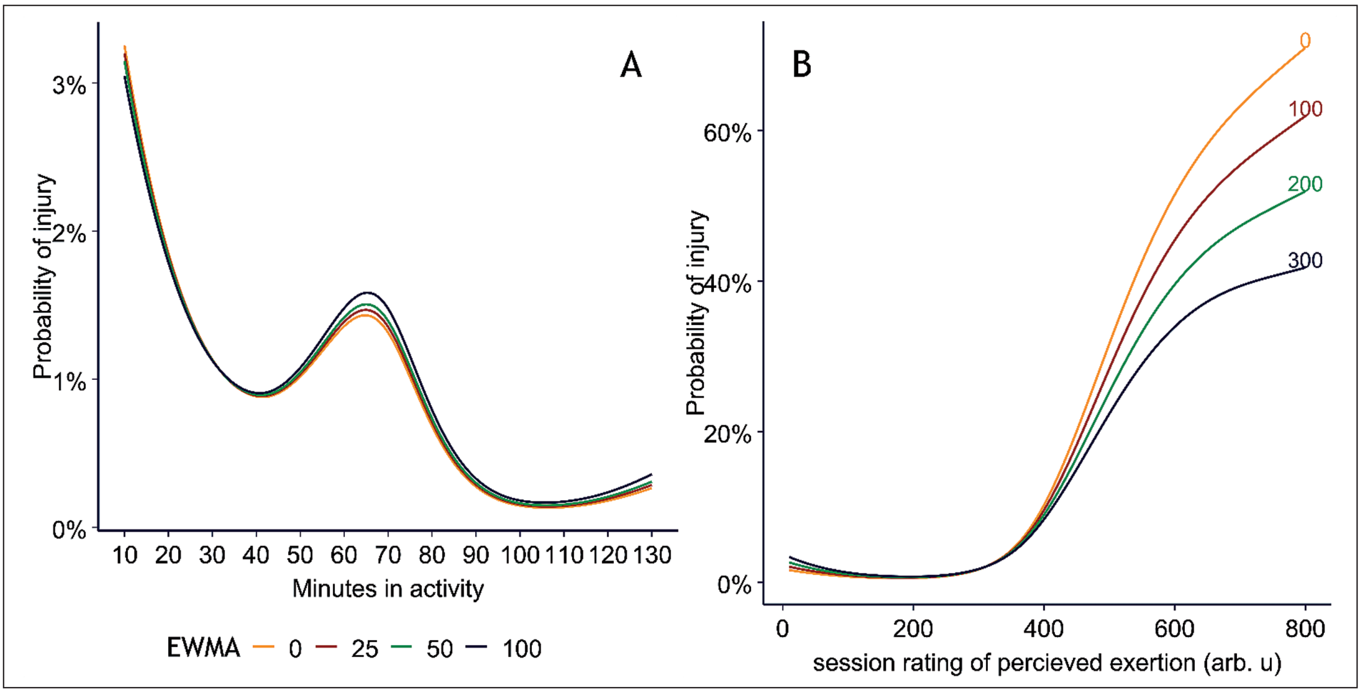

FIG. S4. Probability of injury on the current day (Day 0, acute load) predicted by logistic regression models, using the exponentially weighted moving average to calculate cumulative chronic load. Shown for each level of training load variables used in (A) Qatar Stars League model (420 329 exposure values, 1 977 injuries) and (B) Norwegian elite U-19 model (4 719 exposure values, 60 injuries). The probability is shown for zero, low, medium and high levels of the exponentially weighted moving average. Arb. u = arbitrary units.

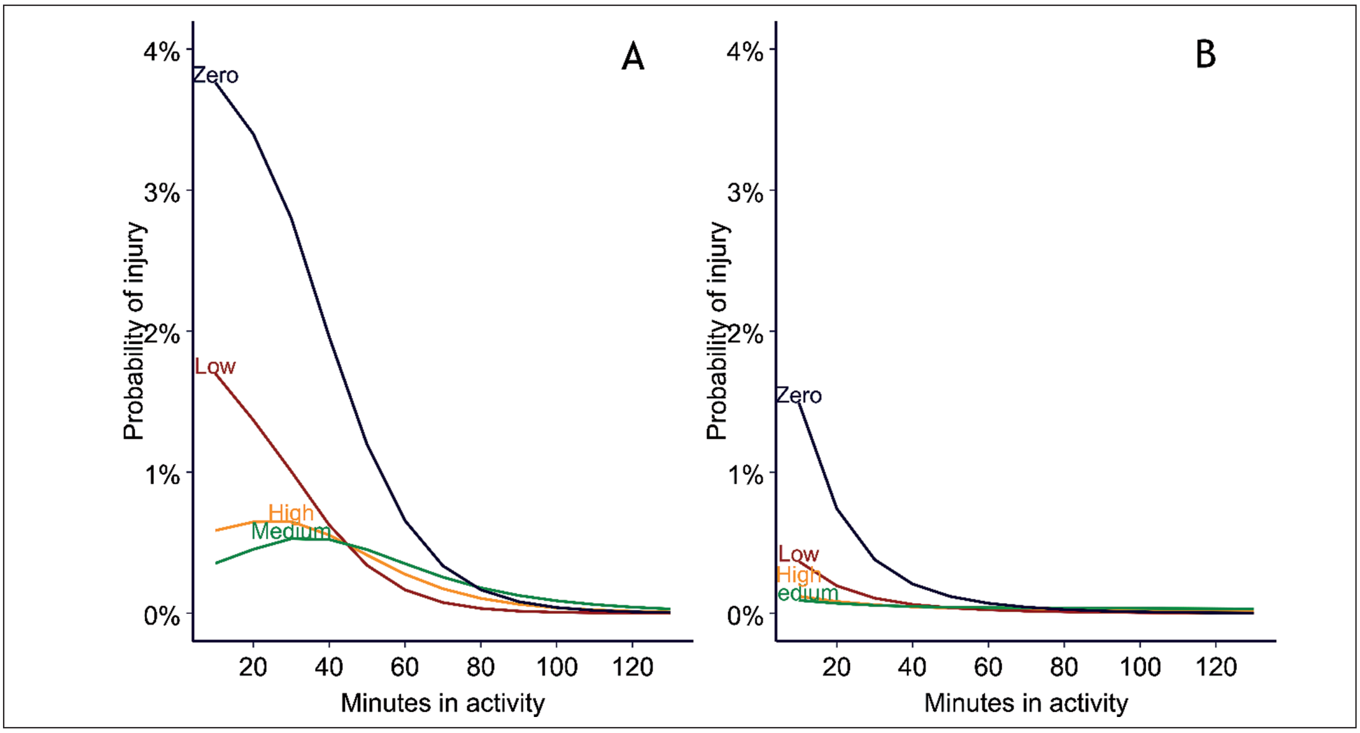

FIG. S5. Probability of injury on the current day (Day 0, acute load) for each minute in activity in the Qatar Stars League population (420 329 exposure values), stratified by (A) sudden onset injuries (n = 1 625) and (B) gradual onset injuries (n = 320). The probability is shown for zero, low, medium and high cumulative chronic minutes in activity. The probability is shown for zero, low, medium and high cumulative chronic training load levels, as defined in Table S2.

REFERENCES

Barzi F, Woodward M. (2004). Imputations of missing values in practice: results from imputations of serum cholesterol in 28 cohort studies. *American journal of epidemiology*, *160*(1), 34–45.

Moons KG, Donders RA, Stijnen T, Harrell Jr FE. (2006). Using the outcome for imputation of missing predictor values was preferred. *Journal of clinical epidemiology*, *59*(10), 1092–1101.

Peters SA, Bots ML, den Ruijter HM, Palmer MK, Grobbee DE, Crouse III JR, O'Leary DH, Evans GW, RaichlenJS , Moons KG. (2012). Multiple imputation of missing repeated outcome measurements did not add to linear mixed-effects models. *Journal of clinical epidemiology*, *65*(6), 686–695.

Van Buuren S. (2018a). *Flexible imputation of missing data*. Chapman and Hall/CRC.

Van Buuren S. (2018b). *Flexible Imputation of Missing Data*. CRC Press. Retrieved 2021-02-12 from https://stefvanbuuren.name/fimd/
